# Septic Endophthalmitis Following Suprachoroidal Cyclosporine Implant Placement in Two Horses With Equine Recurrent Uveitis

**DOI:** 10.1155/crve/7378792

**Published:** 2026-03-27

**Authors:** Milena Sanchez-Garcia, Ria Chalder, Ben Blacklock, Claudia Hartley, Emma Scurrell, Rachel Neto, David Sutton, Richard J. McMullen

**Affiliations:** ^1^ Department of Clinical Sciences, College of Veterinary Medicine, Auburn University, Auburn, Alabama, USA, auburn.edu; ^2^ The Royal (Dick) School of Veterinary Studies, The University of Edinburgh, Midlothian, UK, ed.ac.uk; ^3^ Cytopath Ltd., Ledbury, Herefordshire, UK; ^4^ Department of Pathobiology, College of Veterinary Medicine, Auburn University, Auburn, Alabama, USA, auburn.edu; ^5^ Weipers Centre Equine Hospital, University of Glasgow, Glasgow, UK, gla.ac.uk

## Abstract

This article presents the clinical and histopathological findings from two horses that developed severe postoperative complications following suprachoroidal procedures routinely employed in the management of equine recurrent uveitis (ERU). Case 1 was a 5‐year‐old warmblood stallion that initially received a unilateral suprachoroidal triamcinolone injection, followed later by bilateral suprachoroidal cyclosporine implant (CSI) placement. At 9 weeks postoperatively, the right eye (which had undergone both procedures) developed secondary glaucoma necessitating enucleation. Case 2 was a 14‐year‐old Appaloosa mare that presented 9 days postoperatively with suspected bilateral CSI rejection. Due to poor visual prognosis, the horse was euthanized. Histopathologic examination of all three affected globes demonstrated findings consistent with severe, suppurative endophthalmitis of bacterial etiology.

## 1. Introduction

Equine recurrent uveitis (ERU) is the leading cause of blindness in horses worldwide, with reports suggesting more than 10% of the horse population is affected [1]. The prevalence of ERU in the United States is between 2%–25%, [2] compared with 8%–10% in Europe [3].

Initial treatment of ERU focuses on mitigating the excessive immune response and reducing inflammation through a combination of topical and systemic medications. However, this approach alone is often insufficient, with many horses requiring more invasive treatments to achieve long‐term disease control [4–9]. Among these more advanced therapeutic strategies, suprachoroidal drug delivery has gained popularity as a means of achieving targeted drug administration.

The suprachoroidal space (SCS)—a potential space between the sclera and choroid—provides direct access to the posterior segment, allowing higher local drug concentrations in the choroid, retinal pigment epithelium (RPE), and ciliary body while minimizing exposure of the anterior segment and systemic circulation [10]. This targeted route of administration can enhance therapeutic bioavailability, prolong intraocular drug residence time, and reduce off‐target adverse effects compared with intravitreal, topical, or systemic delivery methods.

Placement of a sustained‐release cyclosporine implant (CSI) within the SCS has become a well‐established component of ERU management, owing to cyclosporine′s ability to suppress T cell activity [11]. The technique was first described by Gilger et al. in 2006 [5], and has since been demonstrated to provide a safe and effective means of delivering cyclosporine to the ciliary body, choroid‐retina, and optic nerve [5, 12]. To date, only a single case of a severe, vision‐threatening complication has been reported following this procedure, in which implant migration into the anterior chamber resulted in uncontrolled uveitis and necessitated euthanasia [13].

This case series describes a previously unreported, vision‐threatening complication following suprachoroidal CSI placement in two horses.

## 2. Case 1

### 2.1. Case History

A 5‐year‐old Warmblood stallion bred in the Netherlands presented to the Royal (Dick) School of Veterinary Studies (R(D)SVS), Edinburgh, United Kingdom, due to suspected ERU in the right eye (OD; ocular dextrus). A complete ophthalmic examination was performed, including slit‐lamp biomicroscopy (Kowa SL‐17 Slit‐lamp, Kowa Co), indirect ophthalmoscopy (Heine Omega 600 Binocular Indirect Ophthalmoscope, Heine Optotechnik) with a 2.2 panretinal condensing lens (Volk Optical), and direct ophthalmoscopy (Heine BETA 200, Heine Optotechnik). Tear production was assessed using the Schirmer tear test‐1 (STT‐1; Schering‐Plough Animal Health), and intraocular pressure (IOP) was measured with rebound tonometry (TonoVet, iCare). Initial ophthalmic examination revealed that both eyes were visual, normotensive (IOP 10–25 mmHg), and had no signs of active or historic uveitis. Continued monitoring was therefore recommended, with reexamination advised should clinical signs recur.

Approximately 3 months later the horse developed blepharospasm OD and was subsequently diagnosed with anterior uveitis. As transportation to the R(D)SVS was not feasible at that time, initial management was instituted at home using topical and systemic anti‐inflammatory therapy. Samples were also taken for a microscopic agglutination test (MAT) on aqueous humor OD and serum, which yielded a negative and positive (1:400) result, respectively; nonsupportive of a *Leptospira*‐associated uveitis. Polymerase chain reaction (PCR) was not performed due to inadequate sample volume.

Although transient improvement was observed, the uveitis subsequently became refractory to medical treatment, prompting referral back to the R(D)SVS for further evaluation and management.

### 2.2. Clinical Finding and Case Management

On reexamination at R(D)SVS, OD exhibited mild blepharospasm, epiphora, low IOP (10 mmHg), dyscoria, aqueous flare, keratic precipitates, and an incipient anterior cortical cataract. The left eye (OS; ocular sinister) had normal findings except for a small number of bullet‐hole chorioretinal lesions.

The horse was hospitalized and anti‐inflammatory treatment escalated to suppress the acute inflammatory response prior to planned suprachoroidal CSI placement. Treatment consisted of intravenous (IV) flunixin meglumine (Meflosyl, Zoetis) 1.1 mg/kg q12h, IV dexamethasone (Dexadreson, MSD Animal Health) 0.1 mg/kg q24h, dexamethasone/polymyxin B sulfate/neomycin sulfate eye ointment (Maxitrol ointment, Novartis Pharmaceuticals) OD q6h, bromfenac sodium sesquihydrate eye drops (Yellox, Bausch & Lomb) OD q4hr, and 1% atropine sulfate (Minims, Bausch & Lomb) OD to effect.

The day after hospital admission, OS developed mild anterior uveitis. Given the presence of a concurrent ipsilateral superficial periocular abrasion, the uveitis was considered most likely traumatic in origin and resolved rapidly with medical management consisting of dexamethasone/polymyxin B sulfate/neomycin sulfate eye ointment q6h.

During hospitalization, the horse experienced a single episode of pyrexia (rectal temperature 38.9°C) noted during routine clinical monitoring. Hematological evaluation revealed a mild neutrophilia (9.41 × 10^9^/L; reference range: 3.0–6.4 × 10^9^/L) and lymphopenia (0.72 x10^9^/l; reference range: 1.30–4.25 × 10^9^/L). All other hematological parameters were within normal limits. The horse returned to normothermia the following day, and no additional diagnostic investigations were undertaken.

Following 1 week of intensive medical treatment, a suprachoroidal injection of 5 mg triamcinolone acetonide (TA; Kenalog, Bristol Myers Squibb Pharmaceuticals) was administered OD using the technique described by Gagnon et al. [9] as a final attempt to further improve the extent of uveitis prior to suprachoroidal CSI placement. Over the subsequent week, the extent of uveitis OD improved sufficiently to permit surgical placement of two suprachoroidal CSIs (Matrix‐Reservoir Biodegradable CSIs, NC State Veterinary Hospital) OD and one OS.

The procedure was performed under general anesthesia by an ECVO‐boarded ophthalmologist using the technique described by Gilger et al. [5]. Prior to implant placement, the eye was aseptically prepared by gently cleansing the periocular skin with sterile gauze soaked in a 1:10 dilution of povidone‐iodine solution. The ocular surface and conjunctival fornices were then thoroughly irrigated with a 1:50 dilution of povidone‐iodine, allowing for 3 min of contact time before flushing with sterile saline. Standard aseptic technique was observed throughout the procedure, including the use of sterile gloves, gowns, patient drape, and surgical instrumentation. No breaches in asepsis were observed during surgery.

### 2.3. Postoperative Management and Complication

Postoperative therapy included oral flunixin meglumine 0.5 mg/kg q12h, PO prednisolone (Equisolon, Dechra) 0.5 mg/kg q24h, oral trimethoprim‐sulfadiazine (Trimediazine Plain, Vetoquinol UK Ltd) 30 mg/kg q12h, topical dexamethasone/polymyxin B sulfate/neomycin sulfate ophthalmic ointment to both eyes (OU; ocular uterque) q6h, topical bromfenac sodium sesquihydrate eye drops OU q4h, and 1% atropine sulfate OD to effect. The horse was discharged 3 days postoperatively on the above medications, with both eyes being comfortable and visual, although low‐grade anterior uveitis persisted OD.

Three weeks following CSI placement, the horse was re‐presented to the R(D)SVS for urgent ophthalmic evaluation due to signs of discomfort OD. Examination revealed anterior and posterior uveitis. The horse was hospitalized, and both topical and systemic anti‐inflammatory therapy intensified. After 1 week of treatment and clinical improvement, the horse was discharged to continue medical management at home, including topical dexamethasone/polymyxin B sulfate/neomycin sulfate ophthalmic ointment OD q6h, topical bromfenac sodium sesquihydrate eye drops OD q6h, flunixin meglumine PO 0.5 mg/kg q12h, and oral prednisolone 0.5 mg/kg q24h. On reexamination 2 weeks later, further reduction in uveitis severity was noted, and the dosing frequency of flunixin meglumine was decreased to once daily.

However, approximately 9 weeks postoperatively, the horse developed signs of ocular pain OD. Assessment at a different referral center closer to the horse′s primary residence (Weipers Centre Equine Hospital, United Kingdom) revealed marked blepharospasm OD, elevated IOP (35 mmHg), corneal edema and neovascularization, and hypopyon. Ocular ultrasonography identified a thickened uveal tract and a large amount of hyperechoic material in the anterior chamber and posterior segment. The eye was subsequently enucleated and submitted for histopathological examination by a board‐certified pathologist. The contralateral eye remained visual, comfortable, and with no active signs of uveitis.

### 2.4. Histopathological Findings

Histopathology of the enucleated globe was consistent with a marked septic (bacterial) suppurative endophthalmitis associated with extensive posterior synechiae, iris bombe, and shallowing of the anterior chamber (Figure 1a). A focal intrascleral pseudocystic cavitation was located adjacent to the ciliary body and peripheral choroid that was consistent with a CSI (Figure 1b). This cavity was surrounded and infiltrated by neutrophilic inflammation associated with multiple bacterial colonies (cocci) (Figure 1b,c). There was marked neutrophilic panuveitis and a large amount of neutrophilic exudate in the vitreous, mixed with multiple large gram‐positive bacterial colonies (cocci) (Figure 1d). Abundant neutrophils coated and infiltrated the ciliary body epithelium, and the nonpigmented ciliary body epithelium occasionally had intracytoplasmic structures that were highly suspicious for hyaline crystals. There was diffuse panretinal atrophy and segmental retinal detachment, and the ciliary cleft and trabecular meshwork were collapsed.

Figure 1Gross and microscopic findings of Case 1. (a) Photograph of the sectioned globe showing marked shallowing of the anterior chamber and abundant vitreous exudate. The black star indicates the site of the CSI. (b) Histopathologic section of the CSI site (hematoxylin and eosin [H&E] stain) demonstrating a cavity formed by the CSI and associated fibrosis. Black arrows indicate bacterial colonies. (c) High‐power view of the bacterial colonies at the CSI site (H&E stain). (d) Gram stain of the vitreous showing numerous gram‐positive cocci.

**Figure:**
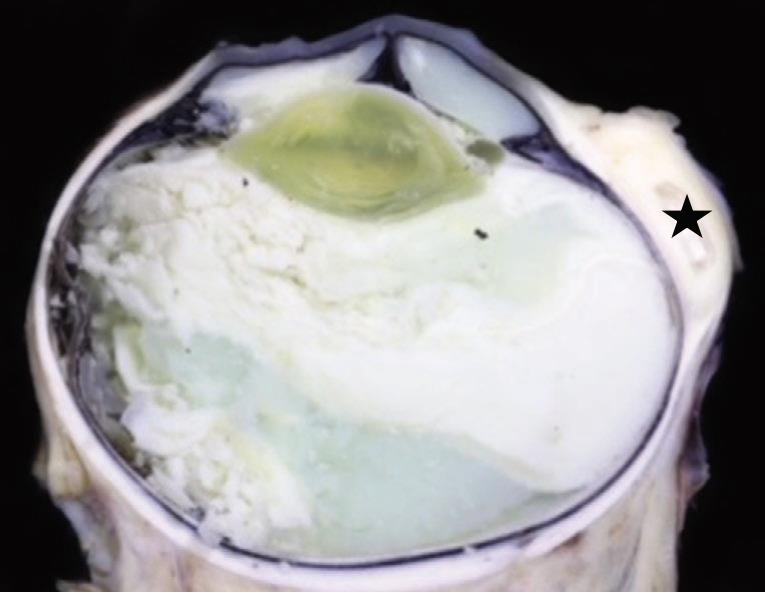
(a)

**Figure:**
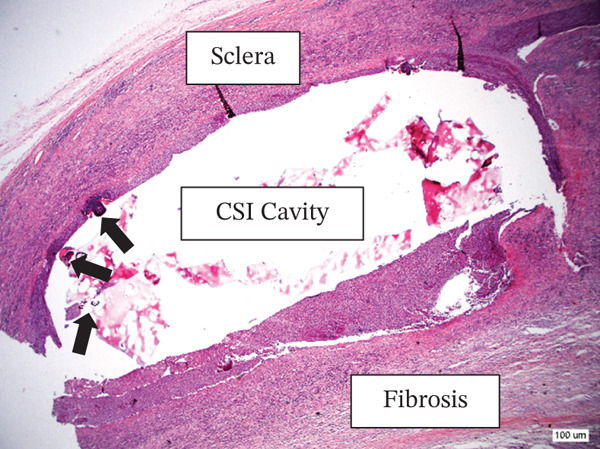
(b)

**Figure:**
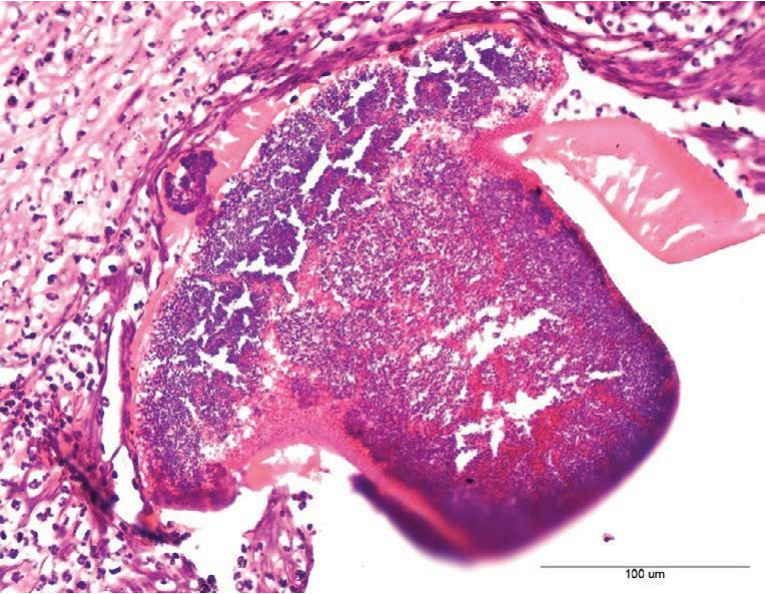
(c)

**Figure:**
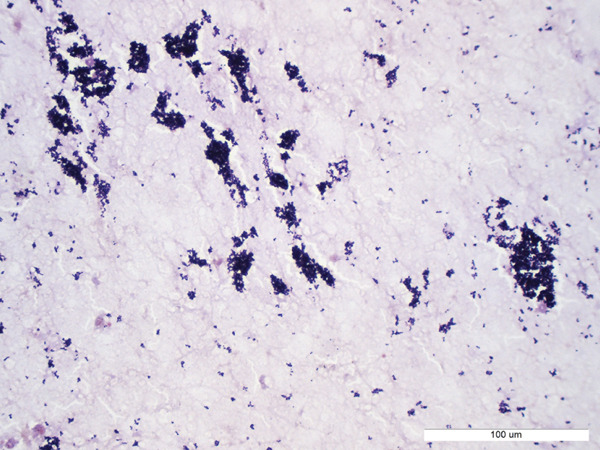
(d)

Using the formalin‐fixed, paraffin embedded (FFPE) tissue, an attempt was made to sequence the 16S rRNA gene using nanopore technology (MinION, Oxford Nanopore Technologies). Unfortunately, this was unsuccessful, likely due to the paraffin embedding of the samples or compromised bacterial integrity, hindering DNA amplification targeting the bacteria ribosomal RNA gene.

## 3. Case 2

### 3.1. Case History

A 14‐year‐old Appaloosa mare with a two‐year history of ERU underwent bilateral suprachoroidal CSI placement by an American College of Veterinary Ophthalmologists (ACVO)‐boarded ophthalmologist at another facility during a quiescent period, using the technique described by Gilger et al. [5].

### 3.2. Postoperative Management and Complications

Information pertaining to preoperative preparation, intraoperative aseptic protocols, and immediate postoperative management was not available for review.

In the days following surgery, OU developed blepharedema and a mucopurulent discharge. Treatment at this stage included terramycin, cefazolin, and silver sulfadiazine cream OU q4h, along with atropine sulfate OU q24h and flunixin meglumine IV (dose and frequency unknown). Four days postoperatively, scleral flap dehiscence was noted OU, and the CSI OS was reported to have spontaneously extruded, whereas the CSI OD was surgically removed. Medical management was continued with the previous therapies, supplemented with oral fluconazole and doxycycline q12h (dosages unavailable).

### 3.3. Clinical Findings and Case Management

The mare presented to Auburn University Veterinary Teaching Hospital (AUVTH) 9 days postoperatively, at which time a complete ophthalmic examination was performed, including slit‐lamp biomicroscopy (Kowa SL‐17, Kowa Co), indirect ophthalmoscopy (Heine Omega 500 Binocular Indirect Ophthalmoscope, Heine Optotechnik) with 14‐D and 20‐D condensing lenses (Volk Optical). Tear production was assessed using the STT‐1 (Schering‐Plough Animal Health) and IOP was measured with rebound tonometry (TonoVet, iCare). The STT‐1 was increased OD and normal OS (35 mm/9 s OD, 31 mm/60 s OS), whilst IOP was low OU (9 mmHg OD, 4 mmHg OS). Marked blepharospasm, hyperemia, and chemosis were present OU, with an absent menace response and dazzle reflex. Variable degrees of posterior synechiae were present, as was a dense area of white‐yellow infiltrate at the surgical site OU, and OD exhibited 360°of corneal vascularization, diffuse corneal edema, keratic precipitates, marked aqueous humor flare and a large fibrin clot in the anterior chamber (Figure 2a,e). In addition, OS exhibited ciliary flush, generalized corneal edema and pigmentation (limiting evaluation of the anterior chamber), and an axial superficial corneal ulcer (Figure 2e). As part of the routine workup for ERU at AUVTH, aqueous humor OU and serum samples were collected and tested for *Leptospira* spp. via MAT and PCR. The aqueous humor obtained was discolored OU, appearing dark yellow OD and dark brown/black OS (Figure 2b,f). The PCR results were negative for all three samples, and MAT results were 1:800 in serum and 1:200 in both aqueous humor samples for *L. Bratislava*. All other *Leptospira* serovars yielded negative results.

Figure 2Clinical, gross, and microscopic findings from Case 2. The stars indicate the site of the CSI. (a) Clinical photograph of OD showing corneal edema and vascularization, fibrin in the anterior chamber, a midrange pupil, and marked yellow discoloration of the ocular fluids. The CSI site is not visible. (b) Aqueous humor obtained following euthanasia was dark yellow and resembled serum. (c) Photograph of the sectioned globe. (d) H&E–stained photomicrograph of OD. (e) Clinical photograph of OS showing a white‐yellow infiltrate at the surgical site dorsally, conjunctival and scleral hyperemia, and generalized corneal edema and pigmentation. (f) The aqueous humor obtained following euthanasia was dark brown/black in color. (g) Photograph of the sectioned globe. (h) H&E–stained photomicrograph of OS.

**Figure:**
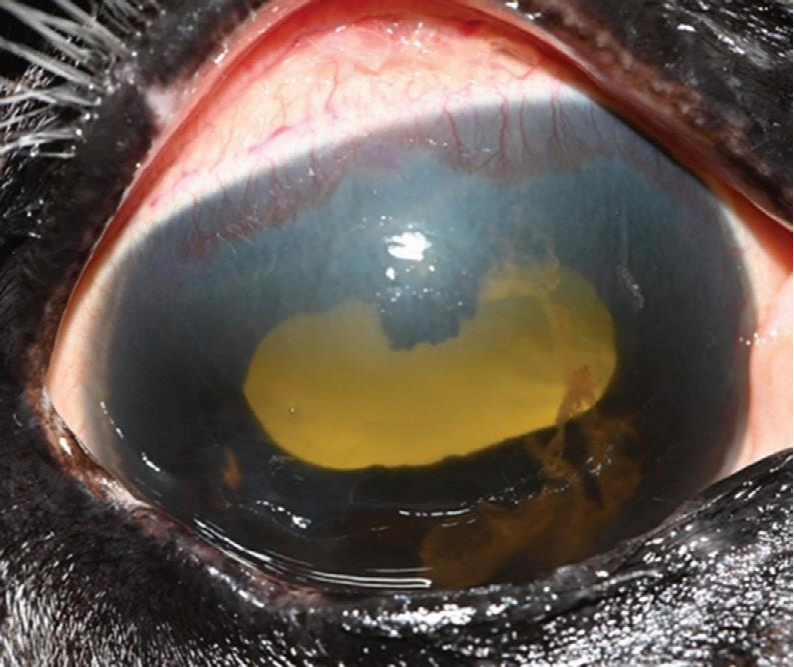
(a)

**Figure:**
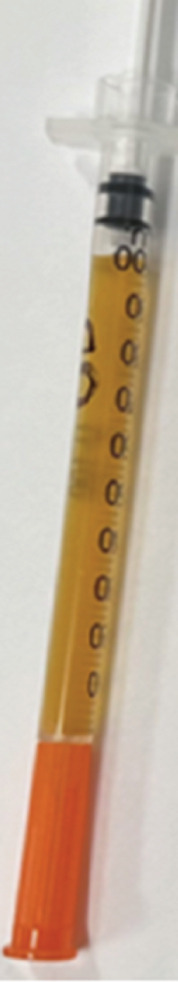
(b)

**Figure:**
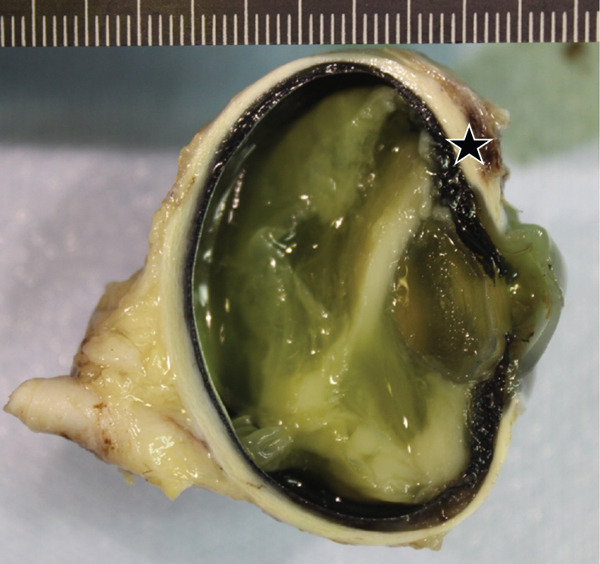
(c)

**Figure:**
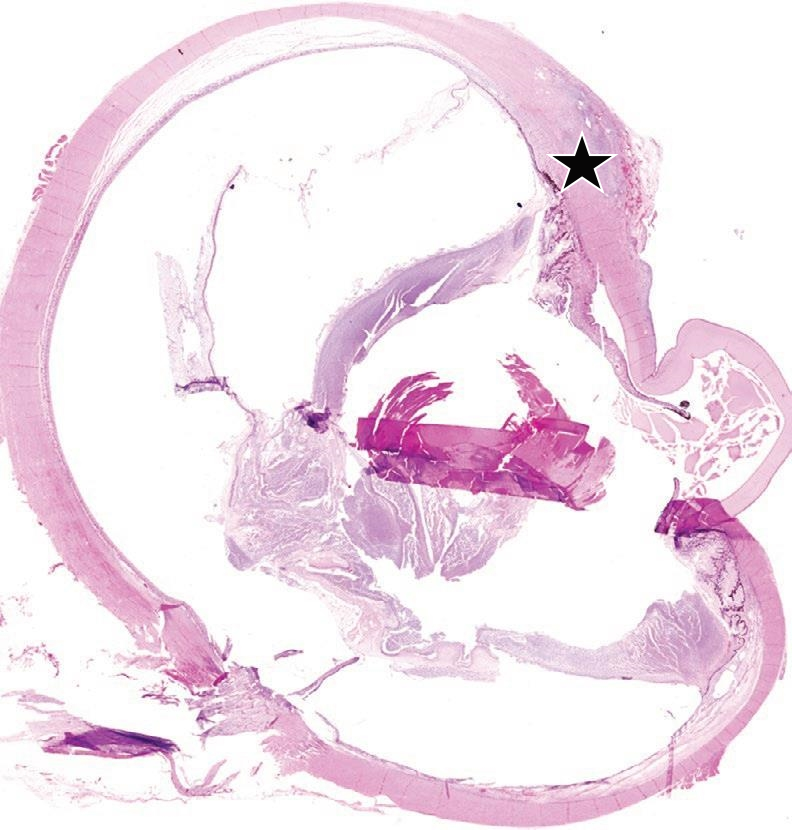
(d)

**Figure:**
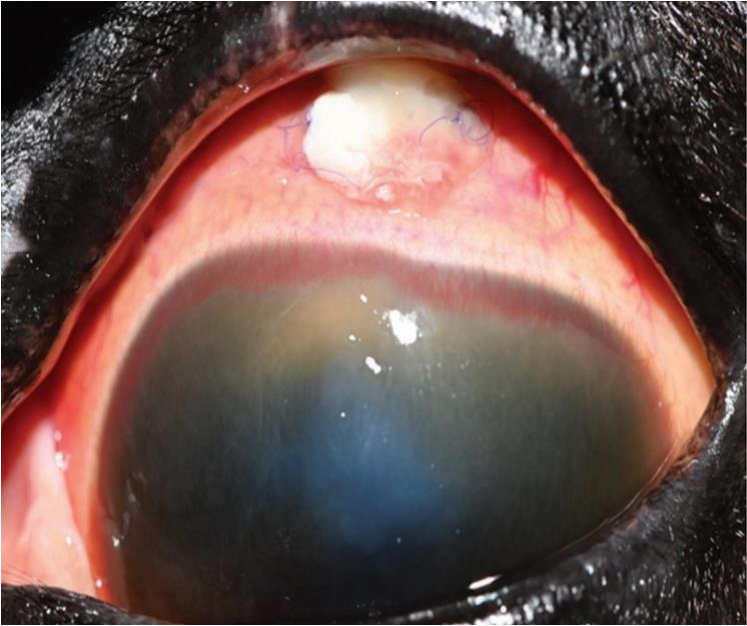
(e)

**Figure:**
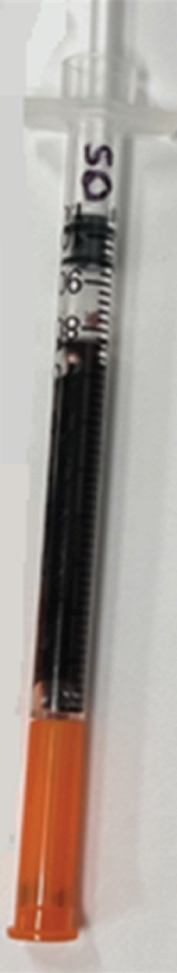
(f)

**Figure:**
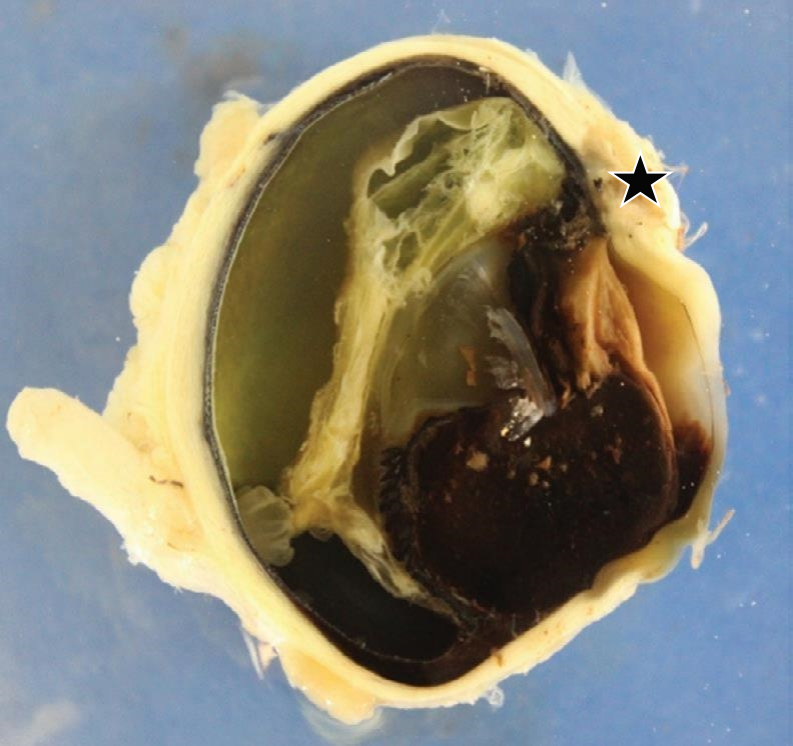
(g)

**Figure:**
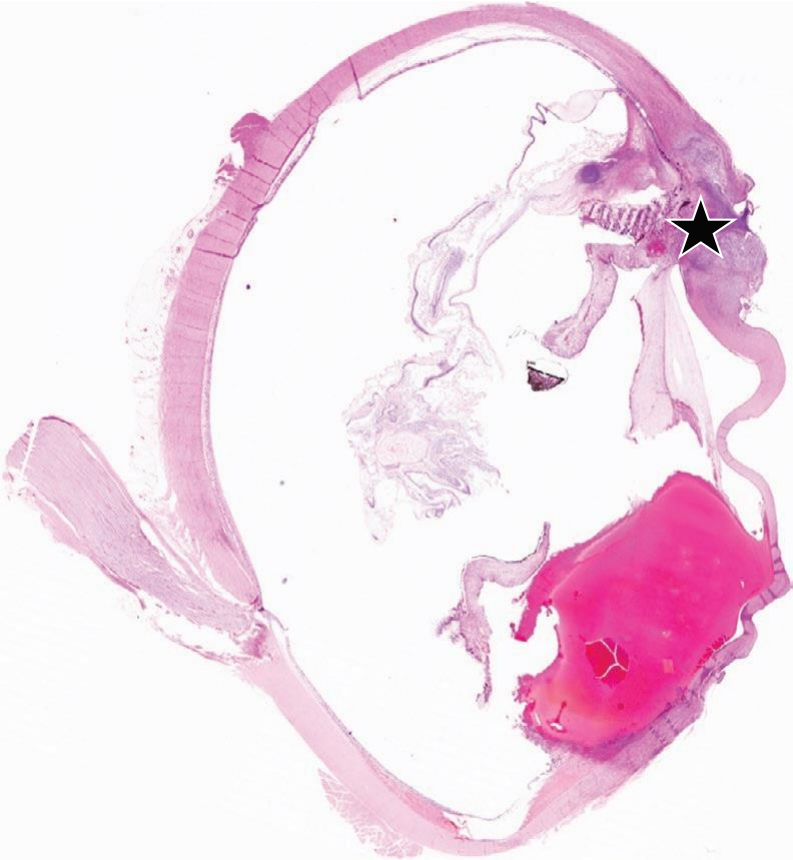
(h)

Transcorneal ocular ultrasonography was also performed, demonstrating a large amount of hyperechoic material within the posterior segments, bilateral exudative retinal detachments (Figure 3a,b), and a posterior lens subluxation OS (Figure 3b).

Figure 3Ultrasonographic images of both eyes from Case 2. The white stars denote the lens. (a) Ultrasonography of OD demonstrated an accumulation of hyperechoic material within the vitreal chamber and an exudative retinal detachment. The lens was in its normal position. (b) Ultrasonography of OS also demonstrated an accumulation of hyperechoic material in the vitreal chamber and an exudative retinal detachment. The lens was posteriorly subluxated.

**Figure:**
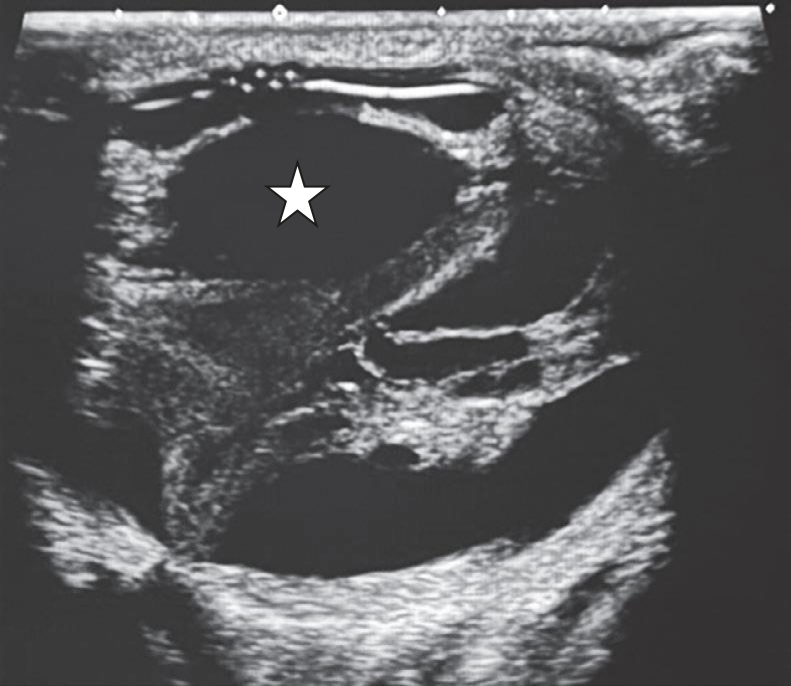
(a)

**Figure:**
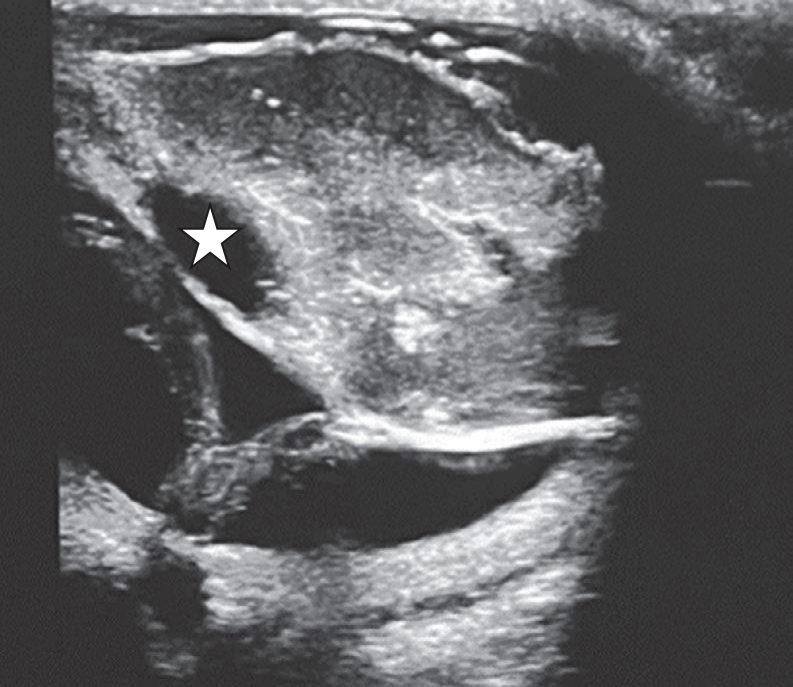
(b)

Electroretinography (OcuScience HMsERG model 2010) revealed minimal to no functional photoreceptor activity OU: OD showed very low‐amplitude a‐ (2.4 uV OD, 15.2 uV OS) and b‐waves (68.3 uV OD, 90.5 uV OS), and significantly prolonged implicit times were associated with both a‐ (73.2 mS) and b‐ (105.5 mS) waves OS. Euthanasia and bilateral enucleation were discussed, with the owners deciding on euthanasia as the preferred option. The protocol for euthanasia included sedation with Xylazine (Rompun, Dechra) 0.5 mg/kg IV, followed by anesthetic induction with Ketamine HCl (Pfizer Inc.) 2.2 mg/kg IV. Once the horse was fully anesthetized, Pentobarbital (Euthasol, Virbac) 79 mg/kg IV was administered. Both globes were removed following euthanasia and submitted for histopathological evaluation by a board‐certified pathologist. Culture and sensitivity analyses of samples from the surgical sites were not conducted due to the mare′s prognosis, the delayed presentation, and prior administration of several days of multiple antimicrobial therapies.

### 3.4. Histopathological Findings

Histopathology of OD demonstrated a thickened dorsal‐peripheral fibrous tunic with associated edema, hemorrhage, and granulation tissue (previous CSI site). A mixture of intact and degenerate neutrophils, lymphocytes, plasma cells, and histiocytes was present within the affected area, and remnants of the suture material were seen in this region (Figure 4). The inflammatory reaction was similar in OS but with more abundant neutrophils, and the area of necrosis was denser. The posterior chamber and anterior to central vitreal cavity were filled with necrotic neutrophils, fibrin, and granular debris OU. Amidst this fibrinosuppurative exudate were intraleukotic small clusters and extracellular larger colonies of gram‐positive cocci (Figure 4c,d). The retina was detached, and some segments of pale eosinophilic vacuolated retinal tissue (marked liquefactive necrosis) were in the posterior segment OU, whereas the optic nerve OD showed evidence of both intramyelinic edema and demyelination. The anterior chamber contained protein‐rich homogeneous exudate (plasmoid aqueous) OU.

Figure 4Photomicrographs of histology sections from Case 2. (a) A dense inflammatory pocket obscured the previous CSI site OS and extended intraocularly, adjacent to the ciliary body. (b) Fibrinosuppurative exudate is seen at the CSI site OD and also surrounding the suture material. (c) Vitreous exudate with colonies of cocci stained with H&E. (d) Colonies of gram‐positive cocci in the vitreal chamber.

**Figure:**
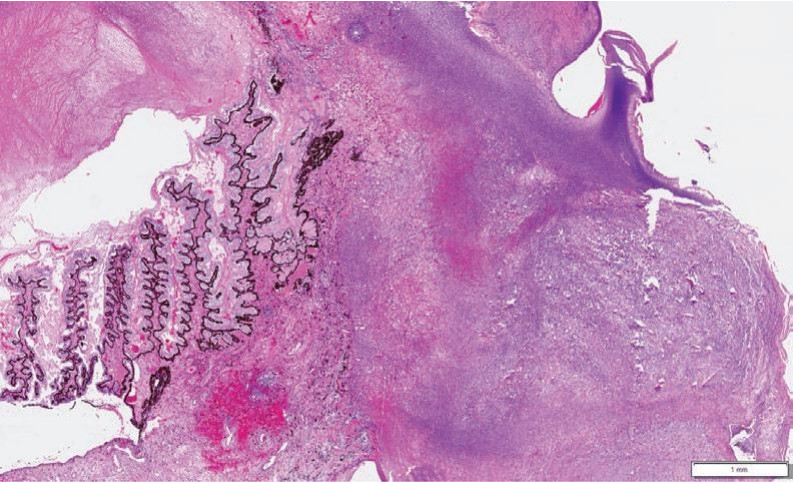
(a)

**Figure:**
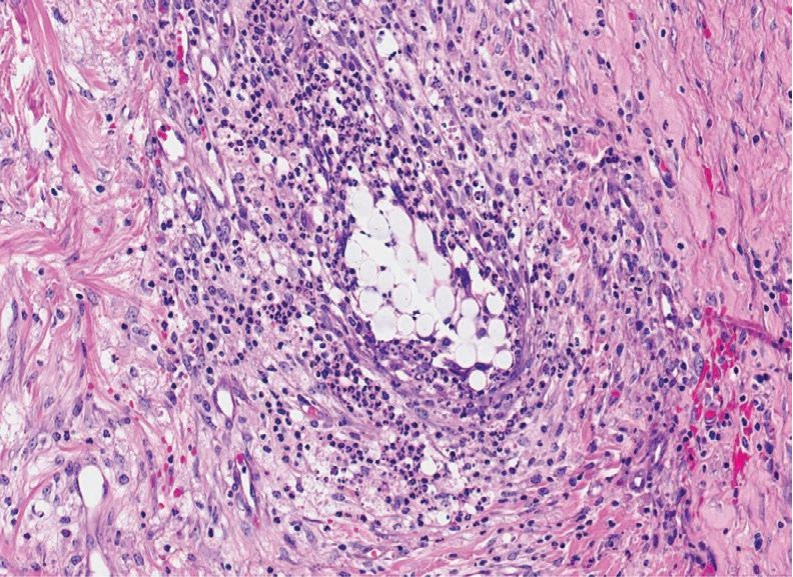
(b)

**Figure:**
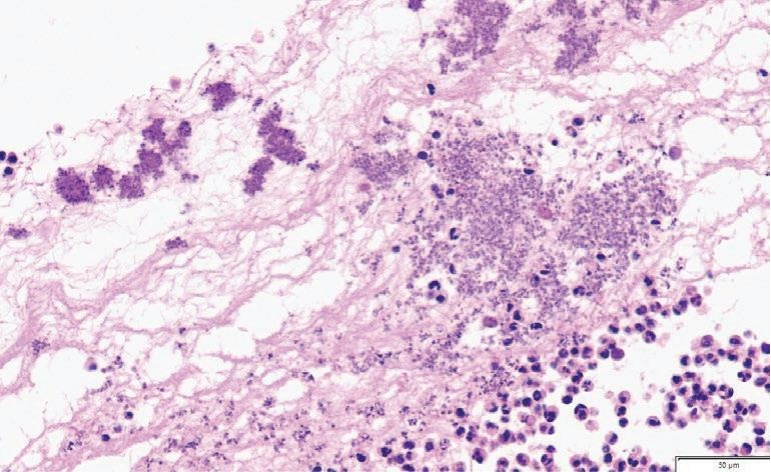
(c)

**Figure:**
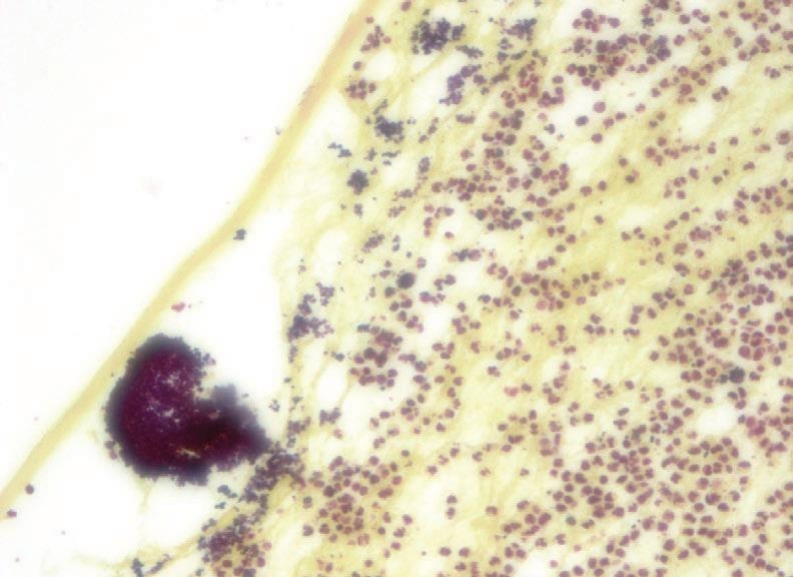
(d)

The anterior uveal stroma contained a moderate lymphoplasmacytic inflammation with amyloid deposits OU (confirmed with Congo red stain). The OD had diffuse corneal stromal edema, dispersed leukocytes, areas of mineralization, peripheral moderate superficial to mid stromal vascularization, and focal Descemet′s membrane rupture. The axial cornea OS had a superficial ulcer. Using the FFPE tissue, samples were sent to the Purdue University Animal Disease Diagnostic Laboratory for attempted DNA amplification and sequencing. The bacterial ribosomal RNA gene was targeted using raw sequence data produced by the Purdue Genomics Core Facility; however, no significant sequence was found.

## 4. Discussion

To date, only two studies have evaluated the safety of suprachoroidal CSIs in horses with ERU [5, 12]. In a study conducted by Gilger et al., CSIs were implanted in six horses with clinically healthy eyes [5]. Postoperative ophthalmic examinations revealed no abnormalities aside from mild conjunctival hyperemia at the implantation site. The horses were euthanized 4 or 9 weeks postimplantation; histologic evaluation of the eyes revealed no pathological changes. A separate arm of the same study followed 67 horses with naturally occurring ERU that underwent suprachoroidal CSI placement and reported postoperative complications of superficial corneal ulceration (three eyes), glaucoma (two eyes), and mild progression of cataract (three eyes) [5] However, no cases of bacterial endophthalmitis occurred [5]. Similarly, a study looking at the long‐term outcomes of CSI placement in horses with naturally occurring ERU reported no such cases in 151 eyes from 133 horses [12].

The application of substance‐release implants in veterinary medicine has been investigated in various animal models in other anatomical locations, including the vitreous in mice [14] and subconjunctival space in rabbits [15], with no postoperative complications reported. In contrast, severe endophthalmitis has been described following placement of intravitreal implants in horses and humans [16, 17].

To the authors′ knowledge, this is the first case series describing bacterial endophthalmitis as a complication of suprachoroidal surgery in horses. Endophthalmitis is characterized by inflammatory infiltration of the intraocular chambers and adjacent tissues, most commonly of bacterial or fungal origin, and constitutes an uncommon but potentially vision‐threatening condition [18, 19]. In veterinary medicine, the majority of cases are exogenous in nature, typically arising secondary to intraocular surgery or a penetrating injury [18]. Conversely, endogenous endophthalmitis results from hematogenous dissemination of infectious agents from a distant site [19].

Given the surgical histories of the presented cases, an exogenous etiopathogenesis is considered most likely. However, an endogenous origin cannot be entirely excluded, particularly in Case 1, which exhibited pyrexia and lymphopenia 1week prior to surgery.

Risk factors for endophthalmitis in horses are not well defined. In human ophthalmology, increased age, gender, immunocompromise, and higher comorbidity index are associated with an increased risk [20–22]. Perioperative factors play a critical role, including adequacy of surgical preparation, intraoperative aseptic technique, operative time, and instrument sterility [22, 23]. Recommended prophylactic measures include the use of sterile gloves and masks (with an emphasis on avoiding speaking during the procedure), minimal manipulation of the eyelids and eyelashes, ruling out adnexa infection, and topical antisepsis [23, 24]. In Case 1, routine surgical preparation was performed prior to the placement of the CSI, with no known breaks in asepsis intraoperatively. In contrast, the surgical site preparation for Case 2 remains unknown, leaving the possibility that inadequate surgical site preparation and/or poor intraoperative aseptic technique played a role in the development of enophthalmitis.

In Case 1, the affected eye had received a TA injection into the SCS 1 week prior to CSI surgery. Although it is unclear whether this injection contributed to the subsequent development of endophthalmitis, it is plausible that it further compromised local immune defense, increasing susceptibility to infection. It should be noted, however, that many horses undergoing CSI surgery for ERU are concurrently receiving topical and/or systemic corticosteroids at the time of surgery and postoperatively without complication. Alternatively, the injection itself could have served as a source of bacterial inoculation. The absence of complications in the contralateral eye, which did not receive a TA injection, further supports the potential involvement of this injection in the pathogenesis of endophthalmitis in Case 1.

Interestingly, the timeframe between surgery and enucleation or euthanasia varied markedly between the two cases. Case 1 underwent enucleation 9 weeks postoperatively, whereas Case 2 was euthanized less than 1 week after surgery due to the development of postoperative complications. In human ophthalmology, a condition termed chronic postoperative endophthalmitis (CPE) is recognized, characterized by an insidious onset of intraocular infection and inflammation occurring more than 6 weeks postoperatively [25]. The causative organisms in CPE are typically of low virulence, with gram‐positive *Cutibacterium* (formerly *Propionibacterium*) and coagulase‐negative *Staphylococcus* spp., accounting for the majority of reported cases. The coccoid morphology of the bacteria observed in Case 1 raises the possibility of infection with a coagulase‐negative *Staphylococcus* species consistent with the chronic disease course. Due to the protracted postoperative interval, such infections are at risk of delayed diagnosis and may be misinterpreted as autoimmune uveitis, leading to inappropriate treatment.

In conclusion, this report documents the first reported cases of bacterial endophthalmitis following suprachoroidal procedures in horses; highlighting important considerations for surgical management and postoperative care. The variable timing and clinical course observed underscore the need for vigilant postoperative monitoring and early recognition of infection.

## Author Contributions

Milena Sanchez‐Garcia and Ria Chalder contributed equally to the writing and preparation of this work and are co‐first authors. Ben Blacklock, Claudia Hartley, Emma Scurrell, Rachel Neto, David Sutton, and Richard J. McMullen Jr. reviewed and edited the document.

## Funding

No funding was received for this manuscript.

## Disclosure

All authors have read and approved the final version of the manuscript. The authors affirm full responsibility for the originality, accuracy, and authorship of the work presented.

## Ethics Statement

Ethical approval was granted for Case 1 through the Royal (Dick) School of Veterinary Studies Veterinary Ethical Review Committee (Reference 68.23). For Case 2, informed consent from the owner was obtained for the examination and subsequent euthanasia. Decisions for performing euthanasia of client‐owned animals at the Auburn University College of Veterinary Medicine occur in collaboration between the attending veterinarian and the owner of the animal. The confidentiality of the patient and owner information was rigorously maintained.

## Conflicts of Interest

The authors declare no conflicts of interest.

## Data Availability

No datasets have been generated or analyzed for this study. All relevant data are included in this article, whereas additional case information is retained in the medical records at The Royal (Dick) School of Veterinary Studies (Case 1) and Auburn University (Case 2). The draft and additional images can be found with the authors.
